# Comment on ‘Myosteatosis and Muscle Loss Impact Liver Transplant Outcomes in Male Patients With Hepatocellular Carcinoma’ by Lu et al.

**DOI:** 10.1002/jcsm.13620

**Published:** 2024-10-20

**Authors:** Aikaterini Kamiliou, Vasileios Lekakis, George Xynos, Evangelos Cholongitas

**Affiliations:** ^1^ First Department of Internal Medicine Laiko General Hospital, Medical School of National and Kapodistrian University of Athens Athens Greece; ^2^ Department of Gastroenterology Medical School of National and Kapodistrian University of Athens, General Hospital of Athens “Laiko” Athens Greece

**Keywords:** cirrhosis, liver transplantation, mortality, myosteatosis, prognosis

**Abbreviations**
CI: confidence interval
HCC: hepatocellular carcinoma
LT: liver transplantation

Dear Editor,

We read with great interest the article by Lu et al. [1] regarding the impact of myosteatosis on post–liver transplantation (LT) outcome in males transplanted for hepatocellular carcinoma (HCC). The authors reported a relatively low prevalence of myosteatosis (27.8% in males) using a gender‐based definition (i.e., muscle attenuation less than 37.5 HU at the third lumbar vertebra of cross‐sectional CT image for men). However, in our recently published meta‐analysis [2] including 10 studies with 3316 HCC patients, the overall pooled prevalence of myosteatosis was estimated as high as 50% (95% confidence interval [CI] 35%–65%) [2]. This discrepancy could be attributed to the fact that Lu et al. [1] included mainly chronic hepatitis B–associated HCC patients, although all patients were Asians. Indeed, our meta‐analysis showed that the prevalence of myosteatosis is significantly lower in Asian HCC patients, compared to the non‐Asian HCC patients (pooled prevalence 45% vs. 69%, respectively, *p* = 0.02), whereas viral‐associated HCC patients have significantly less frequently myosteatosis, compared to those with fatty or alcoholic liver disease–associated HCC (pooled prevalence 49% vs. 65% vs. 86%, respectively, *p* < 0.001). Interestingly, the authors [1] concluded that myosteatosis was not associated with post‐LT outcome in females, although they did not provide which cutoff was used for definition of myosteatosis in this subgroup, although no clear explanation was given for this finding. In addition, they found no association between myosteatosis and hepatic encephalopathy although our meta‐analysis [3] showed that cirrhotic patients with myosteatosis, compared to those without myosteatosis, have more frequently a previous history of hepatic encephalopathy (32% vs. 15%, *p* = 0.04) possibly related with the reduction of skeletal muscle capacity to remove ammonia. Finally, it would be interesting if the authors evaluated the impact of diabetes mellitus, which is an known factor associated with myosteatosis and poor prognosis after LT [3, 4], as well as if they compared the post‐LT outcome of HCC patients with sarcopenia and myosteatosis/isolated myosteatosis versus those with isolated sarcopenia, as recent studies have shown that myosteatosis may be a more important factor, compared to sarcopenia, in the pre‐LT setting of patients without HCC [5].

Nevertheless, Lu et al. [1] confirmed the predictive role of myosteatosis in HCC patients not only in the pre‐LT setting [2] but also in the post‐LT outcome [1], indicating the importance of including assessment of myosteatosis in the process for LT evaluation. However, their results [1] need validation in non‐Asian populations, in which the aetiology of liver disease is more frequently metabolic and alcohol‐related HCC.

## Conflicts of Interest

The authors declare no conflicts of interest.
